# Emergence of EGFR C797S as a resistance mechanism to CLN081 in EGFR exon 20–mutant NSCLC: case report

**DOI:** 10.3389/fonc.2026.1812440

**Published:** 2026-08-06

**Authors:** Basil Dabbah, Roni Gillis, Jaber Salim, Adar Yaacov, Bar Cohen, Areen Abu Remilah, Yehonatan Turner, Noam Asna, Nir Peled

**Affiliations:** 1 Helmsley Cancer Center, Shaare Zedek Medical Center, The Hebrew University, Jerusalem, Israel; 2 Department of Nuclear Medicine, Shaare Zedek Medical Center, Jerusalem, Israel

**Keywords:** acquired resistance, case report, CLN081, EGFR C797S, EGFR exon 20 insertion, liquid biopsy, non–small cell lung cancer

## Abstract

EGFR exon 20 insertion mutations represent a distinct subset of non–small cell lung cancer (NSCLC) with limited sensitivity to earlier-generation EGFR tyrosine kinase inhibitors (TKIs). CLN081 (zipalertinib; TAS6417) is a novel covalent EGFR TKI under development with activity against EGFR exon 20 insertion variants. We report the case of a 44-year-old man with stage IVB lung adenocarcinoma harboring an EGFR exon 20 insertion mutation who received multiple lines of systemic therapy, including CLN081. After eight months of treatment with CLN081, liquid biopsy revealed the emergence of a secondary EGFR C797S mutation (p.Cys797Ser; variant allele frequency [VAF] 0.05%), concurrent with a reduction in the exon 20 insertion allele frequency from 35.1% to 0.5%, accompanied by disease progression. Although C797S-mediated resistance has been hypothesized and demonstrated in preclinical models of CLN081 exposure, this represents, to our knowledge, the first reported *in vivo* identification of an EGFR C797S resistance mutation following CLN081 therapy. This case provides clinically relevant insight into resistance mechanisms associated with emerging EGFR exon 20–targeted therapies.

## Introduction

Activating mutations in the epidermal growth factor receptor (EGFR) occur in approximately 15% of non–small cell lung cancers (NSCLC) in Western populations. Sensitivity to EGFR tyrosine kinase inhibitors (TKIs) varies substantially by mutation subtype. While common mutations in exon 19 and exon 21 confer marked sensitivity to EGFR TKIs, uncommon mutations, including exon 20 insertions, are associated with intrinsic resistance to first-, second-, and third-generation EGFR inhibitors. Structurally, most exon 20 insertions shift the regulatory C-helix into an active-like conformation, narrowing the drug-binding pocket and sterically hindering the access of conventional EGFR TKIs while preserving ATP-binding affinity (1, 2).

EGFR exon 20 insertion mutations account for approximately 4%–9% of EGFR-mutant NSCLC and represent a therapeutically challenging subgroup (3). Amivantamab, a bispecific EGFR–MET antibody, is currently the only FDA-approved agent for this population, while mobocertinib has recently been withdrawn (4). Several investigational agents, including poziotinib, sunvozertinib, and CLN081 (zipalertinib; TAS6417), have demonstrated activity against exon 20 insertions.

Acquired resistance mechanisms to EGFR-targeted therapies include secondary EGFR mutations such as T790M and C797S that abrogates covalent TKI binding, as well as bypass alterations including MET amplification. CLN081 is a covalent EGFR TKI designed to inhibit a broad range of EGFR mutations, including exon 20 insertions and T790M. Phase I/II data from the REZILIENT trial program have demonstrated confirmed objective response rates of 35–41% with CLN081 in previously treated patients with EGFR exon 20 insertion NSCLC (12); however, resistance mechanisms emerging during CLN081 therapy remain poorly characterized in clinical practice, and systematic characterization of acquired resistance in this population has not been reported. We report a case of EGFR C797S mutation emerging after prolonged CLN081 exposure.

## Case presentation

**Figure 1:**
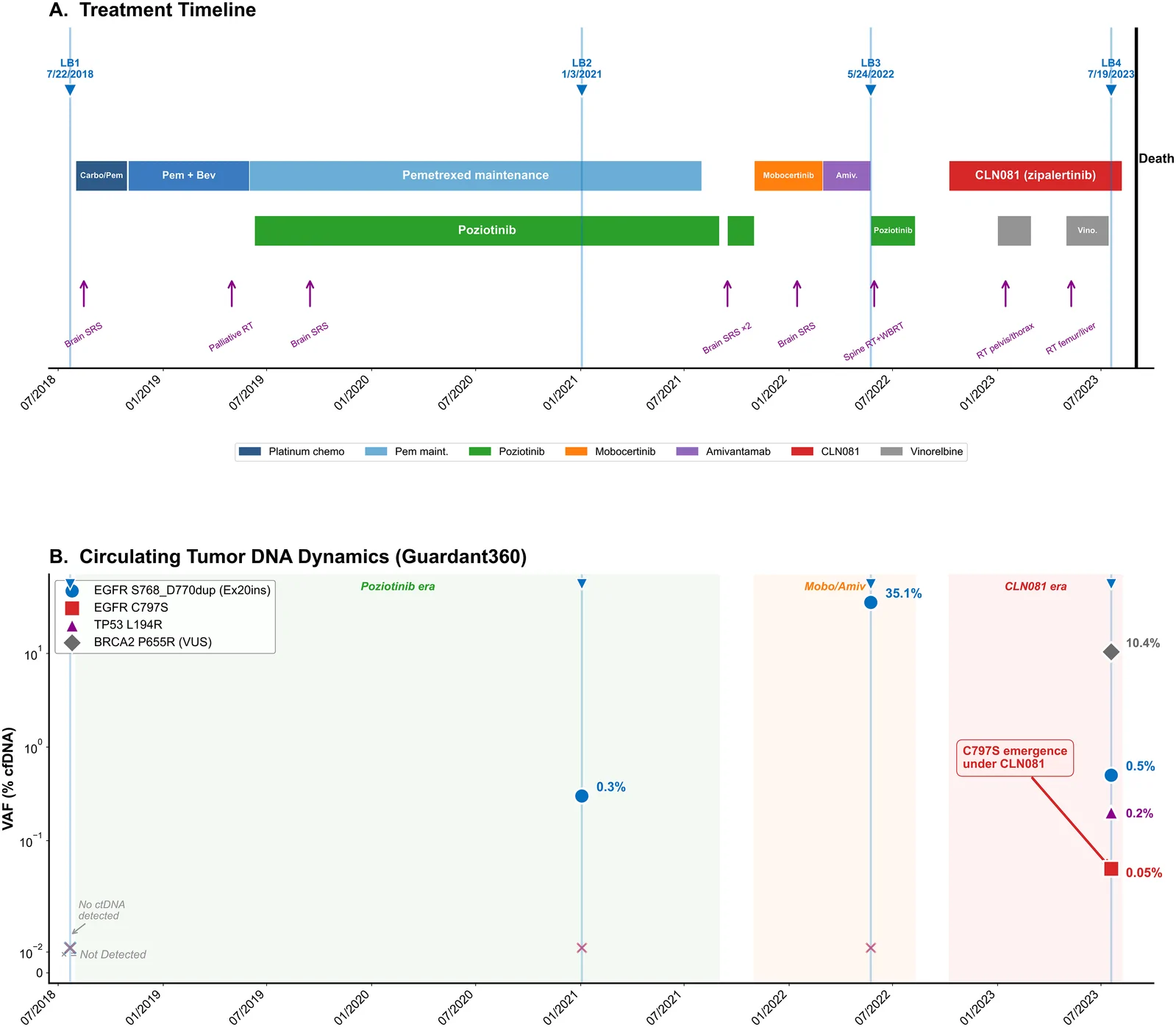
Longitudinal imaging, treatment timeline, and molecular evolution during targeted therapy. **(A)** Serial positron emission tomography–computed tomography (PET-CT) images illustrate disease burden at representative time points during treatment with poziotinib, mobocertinib, amivantamab, and CLN081. The central timeline depicts systemic therapies administered from diagnosis through disease progression. **(B)** Longitudinal liquid biopsy (Guardant360) analysis demonstrates dynamic changes in circulating tumor DNA (cfDNA), including a marked reduction in the EGFR exon 20 insertion (EGFR S768_D770dup) allele frequency following CLN081 initiation and the subsequent emergence of an EGFR C797S mutation. Additional detected alterations are shown with corresponding cfDNA percentages. ND, not detected. Liquid biopsy collection dates (Guardant360): July 22, 2018 (Test 1, no somatic alterations detected); January 3, 2021 (Test 2, EGFR S768_D770dup VAF 0.3%); May 24, 2022 (Test 3, EGFR S768_D770dup VAF 35.1%, CCNE1 amplification 2.2×, EGFR amplification 3.2×); July 19, 2023 (Test 4, EGFR S768_D770dup VAF 0.5%, EGFR C797S VAF 0.05%, TP53 L194R VAF 0.2%, BRCA2 P655R VAF 10.4%). PET-CT images correspond to timepoints A (2020), B (2021), C (2022), and D (2023).

A 44-year-old man with a 45 pack-year smoking history presented with dyspnea and right-sided pleural effusion. Chest and abdominal computed tomography (CT) demonstrated a right pulmonary mass with associated effusion and atelectasis. Cytologic analysis confirmed lung adenocarcinoma. The disease was classified as stage IVB. Programmed death-ligand 1 (PD-L1) expression was less than 1% by immunohistochemistry, ALK rearrangement and ROS1 fusion were negative. Tissue-based comprehensive genomic profiling (FoundationOne CDx) identified an EGFR exon 20 insertion (D770_N771insSVD) as the primary oncogenic driver. Brain magnetic resonance imaging (MRI) revealed a 5-mm intracranial lesion, which was successfully treated with radiotherapy (**Figure 1**).

Initial systemic therapy consisted of pemetrexed (950 mg) and carboplatin (600–500 mg), followed by pemetrexed and bevacizumab (1000–650 mg). After eight treatment cycles, partial radiologic response of the pulmonary lesion was observed. Nine months after diagnosis, liquid biopsy (Guardant360) identified an EGFR S768_D770dup (p.Ser768_Asp770dup) Exon 20 insertion mutation (VAF 0.3%), and poziotinib (16 mg once daily) was initiated. Due to cutaneous and mucocutaneous toxicities, the dose was reduced to alternating 8 mg and 4 mg. Clinical improvement and radiologic response were documented 11 months after diagnosis. During this period, a new corpus callosum lesion was detected and treated with radiotherapy.

Twenty-four months after diagnosis, brain MRI revealed two new cerebral lesions, and positron emission tomography–computed tomography (PET-CT) demonstrated mediastinal and supraclavicular disease progression. Owing to poziotinib toxicity, combination chemotherapy with pemetrexed and carboplatin was reintroduced, followed by pemetrexed monotherapy. Treatment was discontinued due to renal dysfunction (creatinine 1.9 mg/dL), and poziotinib was resumed at 8 mg. Subsequent imaging demonstrated additional cerebral lesions with associated edema.

Thirty-nine months after diagnosis, mobocertinib was initiated at 80 mg once daily and escalated to 160 mg once daily. Imaging revealed progression with bone and multiple intracranial metastases. Due to further renal deterioration, poziotinib was reintroduced briefly before initiation of amivantamab (1050 mg biweekly). After two months, PET-CT and MRI demonstrated progression with new hepatic lesions. Poziotinib, denosumab, and radiotherapy were resumed. Repeat liquid biopsy (Guardant360) revealed no new driver alterations; however, the EGFR exon 20 insertion allele frequency had risen to 35.1%, and transient CCNE1 amplification (2.2×) and EGFR amplification (3.2×) were detected.

Forty-eight months after diagnosis, CLN081 (90 mg twice daily; total daily dose 180 mg) was initiated. Clinical and radiologic improvement was observed, particularly in intracranial disease. After five months, progression of bone metastases prompted vinorelbine therapy. Eight months after CLN081 initiation, liquid biopsy (Guardant360) revealed a marked reduction in the EGFR exon 20 insertion VAF (from 35.1% to 0.5%) and the emergence of a new EGFR C797S mutation (p.Cys797Ser; VAF 0.05%). Additional newly detected alterations included TP53 L194R (p.Leu194Arg; VAF 0.2%) and BRCA2 P655R (p.Pro655Arg; VAF 10.4%, variant of uncertain significance). Microsatellite instability–high status was not detected. Imaging demonstrated widespread progression, and the patient’s clinical condition deteriorated. He died approximately 45 days later.

Radiologic response was assessed by the treating oncologist using serial PET-CT and brain MRI. Formal RECIST 1.1 criteria were not prospectively applied; response was assessed by clinical and radiologic evaluation.

## Discussion

To our knowledge, this is the first reported clinical case demonstrating the emergence of an EGFR C797S mutation following CLN081 therapy in EGFR exon 20 insertion NSCLC. While C797S is a well-established resistance mechanism to osimertinib and other covalent EGFR TKIs, its development after CLN081 exposure has previously been described only in preclinical models (5). Kagawa et al. demonstrated *in vitro* that the C797S substitution conferred resistance to CLN081 in Ba/F3 cells engineered with EGFR exon 20 insertion mutations, establishing a preclinical basis for the clinical observation reported here. Concurrently, clinical data from the REZILIENT Phase I/II program have confirmed the antitumor activity of CLN081 against exon 20 insertions, with confirmed response rates of 35–41% and a manageable safety profile (6, 7); yet, resistance mechanisms in treated patients have remained undescribed. The mechanism involves substitution of cysteine at position 797 with serine, which disrupts the covalent binding of these inhibitors to EGFR, thereby abrogating their inhibitory activity (8).

CLN081 belongs to the class of covalent EGFR tyrosine kinase inhibitors that form an irreversible bond with the thiol group of Cys797 via Michael addition. The C797S substitution replaces this nucleophilic cysteine with serine, eliminating the covalent anchor upon which the inhibitory activity of CLN081 depends. This mechanism is shared across all covalent EGFR TKIs, including osimertinib, mobocertinib, and poziotinib. However, the selective pressure for C797S may vary among agents depending on their binding affinity, kinase selectivity, and the specific conformational context of exon 20 insertion mutations. Notably, a recent analysis of resistance mechanisms to EGFR TKIs in exon 20 insertion NSCLC found that mobocertinib resistance was predominantly mediated by EGFR amplification and secondary on-target mutations rather than C797S (9), suggesting that the emergence of C797S following CLN081 therapy may reflect a distinct resistance trajectory in this population.

The therapeutic landscape for EGFR exon 20 insertion NSCLC has expanded rapidly, with multiple agents representing diverse mechanisms of action. Covalent EGFR TKIs, including CLN081, mobocertinib (now withdrawn), poziotinib, and sunvozertinib, all theoretically carry a vulnerability to C797S-mediated resistance due to their dependence on covalent binding at Cys797. Amivantamab, a bispecific EGFR–MET antibody, acts through a non-covalent mechanism involving receptor internalization and immune effector function, rendering it mechanistically independent of the C797S substitution. Our patient had previously been treated with amivantamab without sustained response, highlighting the complexity of sequential treatment selection. The recently reported REZILIENT-1 data demonstrated reduced efficacy of CLN081 in patients with prior exposure to amivantamab or exon 20–directed TKIs (ORR 14.3%) compared with those treated after platinum-based chemotherapy alone (ORR 40%) (7), underscoring the impact of prior therapy on subsequent treatment efficacy.

This case illustrates the evolutionary pressure exerted by novel exon 20–targeted therapies and highlights the importance of serial molecular monitoring using liquid biopsy (10). The observed reduction in exon 20 insertion allele frequency (from 35.1% to 0.5%) alongside the emergence of C797S (VAF 0.05%) is consistent with on-target resistance as a mechanism of treatment failure. However, this interpretation warrants caution. The very low variant allele frequency of C797S (0.05%) suggests early subclonal emergence, and the concurrent detection of previously unidentified TP53 L194R and BRCA2 P655R alterations raises the possibility of broader genomic instability at disease progression. Furthermore, alternative resistance mechanisms, including tumor heterogeneity, bypass pathway activation (e.g., MET amplification, which was not detected on cfDNA), and histologic transformation, cannot be excluded in the absence of tissue-based analysis at the time of progression. The prior transient detection of CCNE1 and EGFR amplification (May 2022) that resolved under CLN081 therapy further illustrates the dynamic clonal architecture of this tumor under sequential therapeutic pressure. Understanding such resistance pathways will be critical for the development of next-generation inhibitors and rational combination strategies for this difficult-to-treat population.

The emergence of C797S following CLN081 therapy raises important questions regarding subsequent treatment strategies. Fourth-generation non-covalent EGFR inhibitors, including BLU-451, BBT-176, and JIN-A02, are under clinical development and have demonstrated preclinical activity against C797S-containing EGFR mutants by circumventing the requirement for covalent binding (11, 12). EGFR-directed proteolysis-targeting chimeras (PROTACs) represent another emerging strategy, with several compounds demonstrating degradation of C797S-mutant EGFR in preclinical models (12). In the present case, gefitinib (a first-generation reversible EGFR TKI) was added to CLN081 at the time of C797S detection, based on preclinical evidence that C797S in the absence of T790M may retain sensitivity to first-generation inhibitors (13). Prospective molecular surveillance using serial liquid biopsy will be essential for guiding rational sequencing of these emerging therapies.

## Conclusions

We report a case of EGFR exon 20 insertion lung adenocarcinoma in which prolonged treatment with CLN081 was followed by the emergence of an EGFR C797S resistance mutation. This observation provides important clinical insight into resistance mechanisms associated with emerging exon 20–directed EGFR TKIs and underscores the need for ongoing molecular surveillance during targeted therapy. As the therapeutic landscape for EGFR exon 20 insertion NSCLC continues to evolve, characterizing resistance mechanisms to novel agents such as CLN081 will be critical for informing rational treatment sequencing and the development of next-generation inhibitors.

## Data Availability

The datasets presented in this study can be found in online repositories. The names of the repository/repositories and accession number(s) can be found in the article/supplementary material.
